# DETC-based bacterial cellulose bio-curatives for topical treatment of cutaneous leishmaniasis

**DOI:** 10.1038/srep38330

**Published:** 2016-12-06

**Authors:** Fabiana S. Celes, Eliane Trovatti, Ricardo Khouri, Johan Van Weyenbergh, Sidney J. L. Ribeiro, Valeria M. Borges, Hernane S. Barud, Camila I. de Oliveira

**Affiliations:** 1 Instituto Gonçalo Moniz, FIOCRUZ, Salvador, BA, Brazil; 2 Instituto de Química, Universidade Estadual Paulista, Araraquara, SP, Brazil; 3 Universidade de Araraquara-UNIARA, Araraquara, SP, Brazil; 4 Rega Institute for Medical Research, Department of Microbiology and Immunology, K. U. Leuven, Belgium; 5 Instituto de Investigação em Imunologia (iii), INCT, São Paulo, Brazil

## Abstract

The treatment of leishmaniasis still relies on drugs with potentially serious adverse effects. Herein, we tested a topical formulation of bacterial cellulose (BC) membranes containing Diethyldithiocarbamate (DETC), a superoxide dismutase 1 inhibitor. *Leishmania*-infected macrophages exposed to BC-DETC resulted in parasite killing, without pronounced toxic effects to host cells. This outcome was associated with lower SOD1 activity and higher production of superoxide and cytokine mediators. Topical application of BC-DETC significantly decreased lesion size, parasite load and the inflammatory response at the infection site, as well as the production of both IFN-γ and TNF. Combination of topical BC-DETC plus intraperitoneal Sb^v^ also significantly reduced disease development and parasite load. The leishmanicidal effect of BC-DETC was extended to human macrophages infected with *L. braziliensis*, highlighting the feasibility of BC-DETC as a topical formulation for chemotherapy of cutaneous leishmaniasis caused by *L. braziliensis*.

Leishmaniasis is a widespread group of parasitic diseases caused by protozoa of the genus *Leishmania*. Currently, about 12 million people are at risk of leishmaniasis and there are an estimated 1.5–2 million new cases each year^1^. There are two main clinical manifestations: Visceral Leishmaniasis (VL), affecting mainly the spleen and liver, and Cutaneous Leishmaniasis (CL), affecting the skin. CL caused by *Leishmania braziliensis* is distinguished from other leishmaniasis by its chronicity, latency and tendency to metastasize in the human host^2^. Brazil along with nine other countries account for 70–75% of the global CL incidence^3^. First choice drugs for leishmaniasis chemotherapy are pentavalent antimonials (Sb^v^) [Meglumine Antimoniate (Glucantime^®^) and Sodium Stibogluconate (Pentostam^®^)]^4^ which are significantly toxic and with reported drug resistance^5^. Amphotericin B^6^ and Miltefosine^7^ are also limited with regards to toxicity, cost and/or time of treatment, reinforcing the need for new chemotherapeutic alternatives.

Dysregulation of the Superoxide Dismutase 1 (CuZnSOD/SOD1) - superoxide axis has been identified as key problem in CL^8^ and SOD1 plasma levels can predict therapeutic failure in CL caused by *L. braziliensis*^9^. Pharmacological inhibition of CuZnSOD/SOD1 with Diethyldithiocarbamate (DETC), a copper chelator that targets SOD1^10^, significantly reduced *Leishmania* infection *in vitro*^8^ as a result of increased superoxide levels. *In vivo*, intraperitoneal injection of DETC inhibited CL development in *L. braziliensis-*infected mice^11^, indicating the feasibility of treating CL by targeting SOD1-associated pathways.

In the present work, we explored a topical formulation for CL treatment consisting of bacterial cellulose (BC) membranes loaded with DETC. BC membrane is a nanomaterial produced by bacteria in the form of a gel-like membrane^12^,^13^ it has high water content (about 99%) and displays good mechanical properties^14^. BC is biocompatible, permeable to gas and liquids, it improves wound and burn healing and is easily managed by the patient (rev. in ref. 15). Never dried BC’s nanometric dimension and liquid absorption/release capability enable its use as a support for drug release in topical systems^16^,^17^, mainly because of its membranous form. We show that dried BC membranes loaded with DETC (BC-DETC) significantly reduced *L. braziliensis*-infection rate *in vitro* and lesion development *in vivo*. Combination of topical BC-DETC with intraperitoneal Sb^v^ was even more effective against CL development, indicating the feasibility of such approach.

## Results

### Characterization of BC-DETC membranes by SEM and FTIR

**Figure 1:**
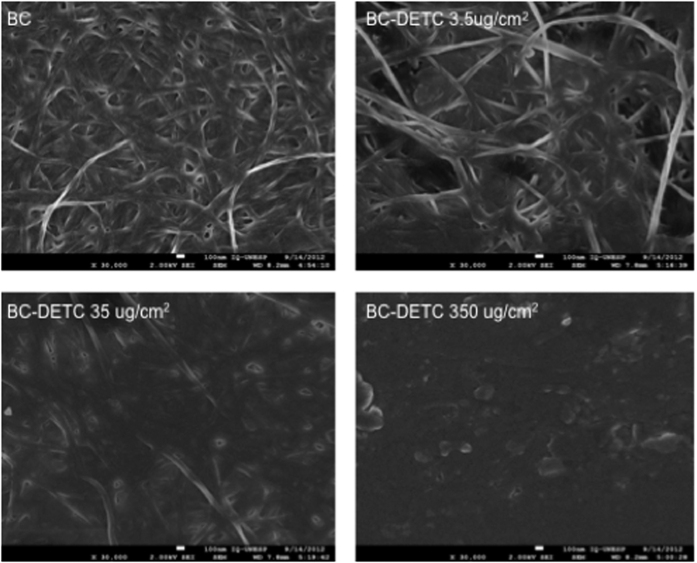
Scanning electron micrographs of bacterial cellulose membrane (BC) and BC membranes containing DETC. All images were obtained on 30,000X magnification.

Initially, we characterized the control (empty) and BC-DETC membranes by SEM. Micrographs showed the characteristic tridimensional fibrillar network of bacterial cellulose (Fig. 1). Upon addition of DETC (3.5 μg DETC/cm^2^), the network does not show the presence of DETC aggregates, indicating homogeneous dispersion without irregular precipitation during compound adsorption onto BC matrices. Also, an increasing density of the DETC was observed in BC networks when higher concentrations of DETC were used (35 μg/cm^2^ and 350 μg/cm^2^) (Fig. 1). BC-DETC FTIR spectra obtained with different concentrations of DETC showed a superposition of both BC and DETC spectrum (Supplementary Figure 1). We observed a shift of two peaks of DETC, from 1673 and 1616 cm^−1^ to 1739 and 1630 cm^−1^, respectively which suggests the non-bonded interaction of oxygen form hydroxyl groups of BC with sulfur from DETC.

### BC-DETC membranes reduce the parasite load in a dose-dependent manner

**Figure 2:**
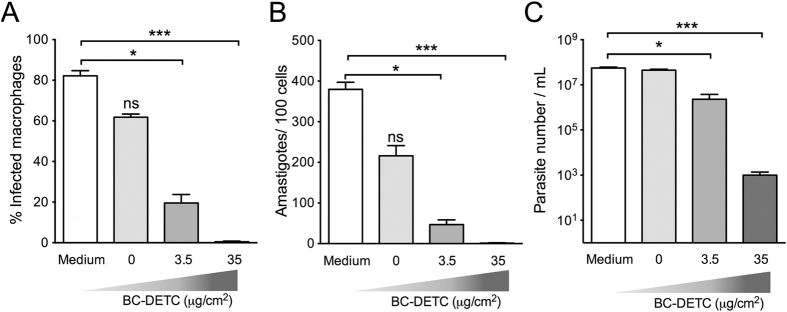
Dose-dependent effect of BC-DETC on *L. braziliensis*-infected macrophages. Macrophages were infected and exposed to empty BC or to BC-DETC (3.5 or 35 μg/cm^2^). Cells were assessed for (**A**) the percentage of infected macrophages and (**B**) the number of amastigotes per 100 macrophages. (**C**) The number of viable parasites was evaluated by culture in Schneider medium, free of BC-DETC. Data are from a representative experiment performed in quintuplicate. *p < 0.05, ***p < 0.001.

Exposure of murine macrophages to BC-DETC at 3.5 or 35 μg DETC/cm^2^ did not change cell viability whereas BC-DETC at 350 μg DETC/cm^2^ reduced cell viability by 40% (Supplementary Figure 2). To evaluate the killing potential, *L. braziliensis*-infected BMDM were exposed to BC-DETC. BC-DETC (3.5 and 35 μg DETC/cm^2^) significantly decreased the number of infected macrophages (Fig. 2A) and the number of amastigotes per 100 cells (Fig. 2B). Exposure to empty BC (0 μg DETC/cm^2^) did not decrease *L. braziliensis* infection in a significant manner (Fig. 2A and B). To confirm that DETC compromised parasite viability, intracellular parasite survival was quantified by transformation of amastigotes into proliferating promastigotes in Schneider’s medium, as described^8^. *L. braziliensis* promastigotes were significantly reduced following exposure of infected BMDM to BC-DETC (Fig. 2C) but not upon exposure to empty BC. These results indicate that DETC is acting on infected macrophages, leading to parasite killing.

### BC-DETC reduces SOD activity and increases superoxide and cytokine release in *L. braziliensis*-infected murine macrophages

**Figure 3:**
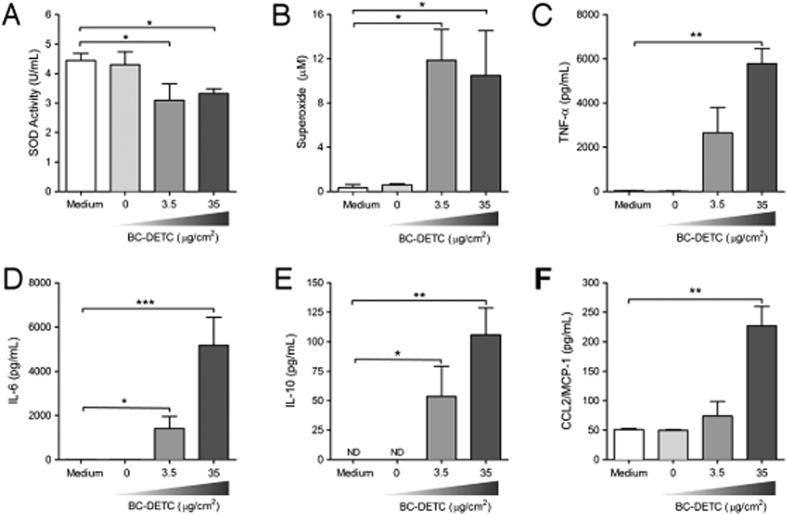
BC-DETC treatment reduces SOD activity and modulates cytokine release in *L. braziliensis*-infected macrophages. Macrophages were infected and exposed to empty BC or to BC-DETC (3.5 or 35 μg/cm^2^). (**A**) SOD activity, (**B**) superoxide, (**C**) TNF, (**D**) IL-6, (**E**) CCL2/MCP-1 and (**F**) IL-10 levels were evaluated after 48 hours. Data are from a representative experiment performed in quintuplicate. *p < 0.05, **p < 0.01, ***p < 0.001.

SOD1 catalyzes the dismutation of superoxide into oxygen and hydrogen peroxide and DETC is a copper chelator that inhibits SOD1, thereby increasing superoxide levels. We therefore evaluated SOD1 activity and superoxide production in *L. braziliensis*-infected macrophages exposed to BC-DETC. SOD1 activity was significantly decreased in BC-DETC-exposed cells (Fig. 3A) whereas superoxide levels were significantly increased in this same condition (Fig. 3B), compared to unexposed controls. Such results are in line with the observed parasite killing upon exposure to BC-DETC (Fig. 2). In the presence of BC-DETC, TNF, IL-6, IL-10 and CCL2 levels were significantly increased (Fig. 3C–F, respectively), an effect not observed in cultures exposed to empty BC (Fig. 3C–F). These results suggest that parasite killing is resulting from increased superoxide levels and SOD1 inhibition by DETC. Increased cytokine production appears to be a side-product of parasite killing by DETC-exposed cells.

### Topically-applied BC-DETC reduces the lesion size and the parasite load *in vivo*

**Figure 4:**
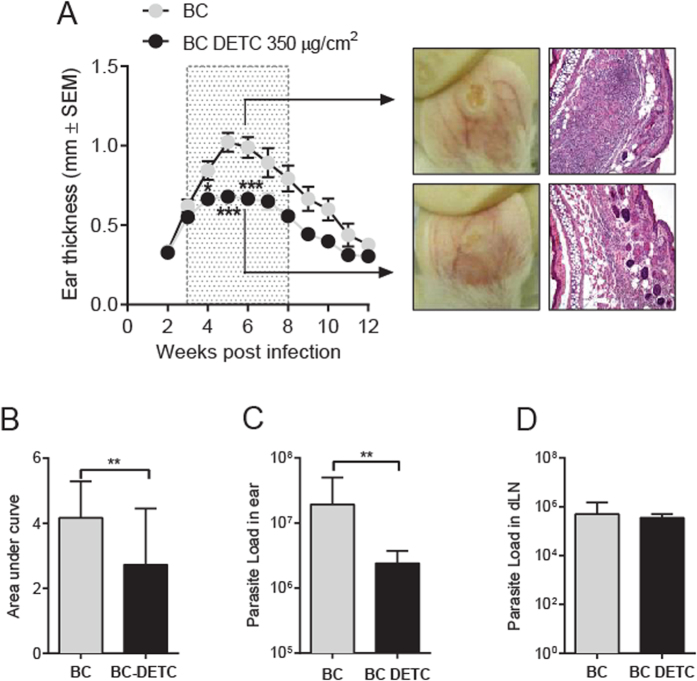
Topical treatment with BC-DETC controls CL development. Mice were infected with *L. braziliensis* and three weeks later BC-DETC (at 350 μg/cm^2^) was applied for three weeks (boxed area). Controls received empty BC. (**A**) Lesion development was measured weekly. Ear sections, obtained six weeks after infection, were analyzed by optical microscopy under 20X magnification. (**B**) Disease burden [shown as Area Under the Curves (AUC) depicted in (**A**)] in mice treated with BC-DETC or empty BC. Parasite load was determined at the infection site (**C**) and at the dLN (**D**), at six weeks, by limiting dilution analysis. Data are pooled from three independent experiments, each performed with four to six mice per group. **p < 0.01.

Next, we tested the effect on BC-DETC *in vivo*, employing an experimental model of CL^18^. Mice were inoculated with *L. braziliensis* and three weeks later, BC-DETC (loaded at 35 and 350 μg DETC/cm^2^) was applied to cutaneous lesions, membranes were changed three times a week. BC-DETC loaded with 35 μg DETC/cm^2^ did not significantly reduce ear thickness in infected mice whereas treatment with 350 μg DETC/cm^2^ induced a significant effect two weeks after the onset of treatment (five weeks after parasite inoculation) (Supplemental Figure 3). Additionally, we did not observe differences comparing mice treated with emtpy BC. Based on this, control mice were treated with empty BC in subsequent experiments. Overall, topical application of BC-DETC (at 350 μg DETC/cm^2^) significantly decreased ear thickness, an effect not observed in control mice (treated with empty BC) (Fig. 4A). In control mice, dermal lesions were visually larger (at six weeks after infection). Histopathological examination of tissue sections showed the presence of an intense inflammatory infiltrate, containing epidermal hyperplasia and ulcerated areas, characteristic of CL lesions^19^ (Fig. 4A). On the contrary, ears of animals treated with BC-DETC showed smaller lesions and a mild cellular infiltrate and less hyperplasia (Fig. 4A). At seven weeks, lesions began to heal spontaneously in both BC-DETC and control groups, as previously described for this experimental model of CL^18^. Disease burden, calculated by the area under the curve (AUC) obtained for mice treated with BC-DETC or with empty BC was also significantly different (Fig. 4B), confirming that BC-DETC inhibits lesion development, *in vivo*. Moreover, topical application of BC-DTEC significantly decreased the parasite load (Fig. 4C), measured six weeks after parasite inoculation (three weeks after the onset of treatment), differently from control mice (treated with empty BC). At this same time point, however, parasite load within draining lymph nodes was similar for BC-DETC and control mice (Fig. 4D). These data demonstrate that topically applied BC-DETC controls lesion development and reduces the parasite load at the lesion site.

**Figure 5:**
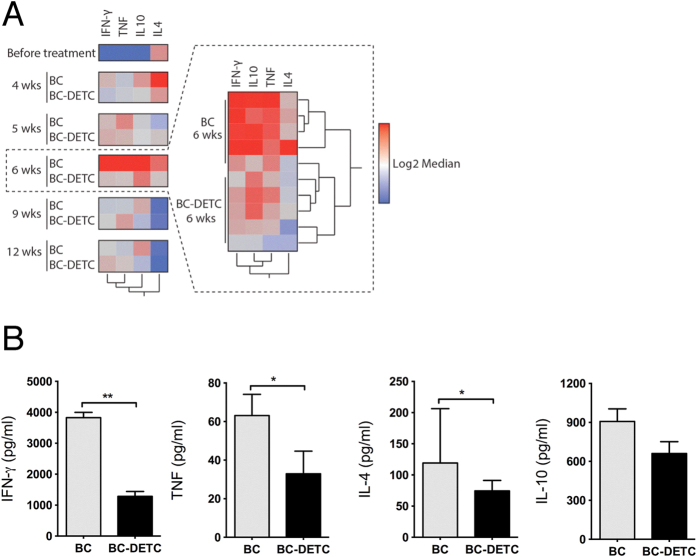
BC-DETC treatment modulates the cellular recall response. Mice were infected and three weeks later BC-DETC (at 350 μg/cm^2^) was applied for three weeks. Controls received empty BC. Draining lymph nodes were re-stimulated *in vitro* and cytokines were quantified by ELISA. (**A**) Heat map depicting cytokine production, colored to indicate levels. Insert shows data for individual mice at six weeks. (**B**) Cytokine levels detected in culture supernatants, at six weeks. Data are from a representative experiment performed with six mice per group. *p < 0.05.

IFN-γ and TNF are associated with the inflammatory nature of the CL lesion^20^ so we also addressed the cellular immune response in mice treated with BC-DETC. Mice were euthanized at different time points during the treatment period and the recall response of lymph node cells was evaluated. IFN-γ, TNF, IL-10 and IL-4 levels were overall higher in control mice compared to BC-DETC-treated mice (Fig. 5A) (Supplemental Figure 4). At six weeks post infection (three weeks after the onset of treatment), the pattern of cellular response in BC-DETC-treated mice was clearly distinct from that observed in control mice (Fig. 5A), corroborating the phenotypic differences observed earlier regarding lesion size and parasite load (Fig. 4A). At this time point, levels of IFN-γ, TNF and IL-4 were significantly lower in mice treated with BC-DETC (Fig. 5B), a finding than can be associated with the milder inflammatory reaction observed (Fig. 4A). Collectively, our results show that topical application of BC-DETC reduces CL lesions and parasite load and, in parallel, modulates the inflammatory response.

### Combination therapy

**Figure 6:**
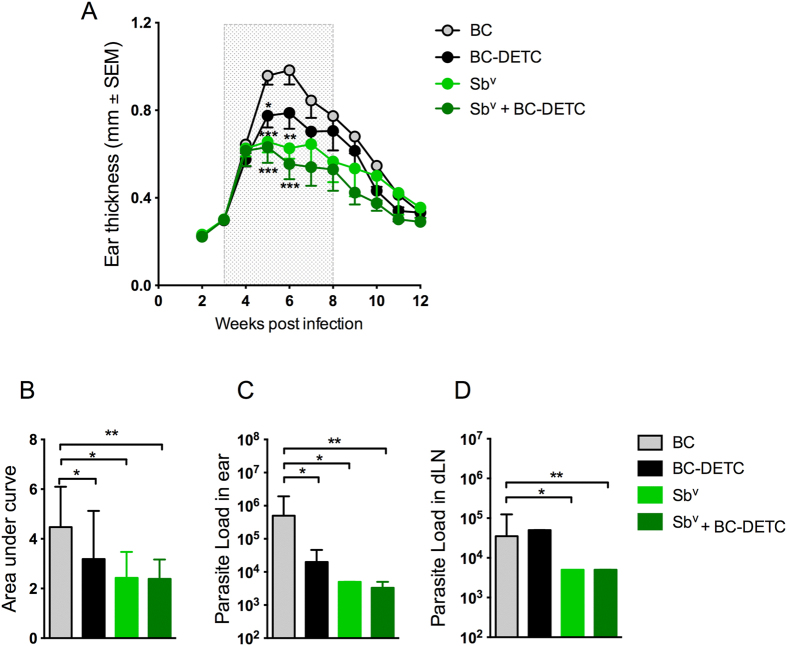
Efficacy of topical BC-DETC and intraperitoenal Sb^v^ combined therapy in *L. braziliensis* infection. Mice were infected and three weeks later were treated with topical BC-DETC (at 350 μg/cm^2^), intraperitoneal Sb^v^ (100 mg/kg/day) or both. Controls received empty BC. (**A**) The course of lesion size development was measured weekly. (**B**) Disease burden [shown as Area Under the Curves (AUC) depicted in (**A**)] for each group. Parasite load was determined at the infection site (**C**) and at the dLN (**D**), 6 weeks after infection, by limiting dilution analysis. Data are from a representative experiment performed with twelve mice per group. *p < 0.05; **p < 0.01; ***p < 0.001.

The drugs of first choice for the chemotherapy of leishmaniasis are pentavalent antimonials (Sb^v^). We therefore investigated whether a combination treatment consisting of topical BC-DETC and intraperitoneal Sb^v^ would result in a more effective control of experimental CL. Mice were infected with *L. braziliensis* and treated with BC, BC-DETC, Sb^v^ or BC-DETC plus Sb^v^. Both Sb^v^ alone and BC-DETC alone significantly reduced lesion size, observed five and six weeks post infection (Fig. 6A) whereas combination therapy consisting of BC-DECT plus Sb^v^ reduced lesion size by approximately 43%, compared to controls. These results were corroborated by the AUC analysis: disease burden in BC-DETC, Sb^v^ and BC DETC plus Sb^v^ were significantly reduced compared to control mice (treated with empty BC) (Fig. 6B). Parasite load was also significantly reduced in the three experimental groups compared to controls (Fig. 6C). Combination treatment significantly reduced parasite load in the draining lymph nodes (Fig. 6D), an effect also observed in mice treated with Sb^v^ alone. Combination therapy consisting of topical BC-DETC plus Sb^v^ reduces CL development *in vivo*; however, we did not observe significant differences compared to treatment with either BC-DETC alone or Sb^v^ alone.

### BC-DETC membranes promotes parasite killing in human macrophages infected with *L. braziliensis*

**Figure 7:**
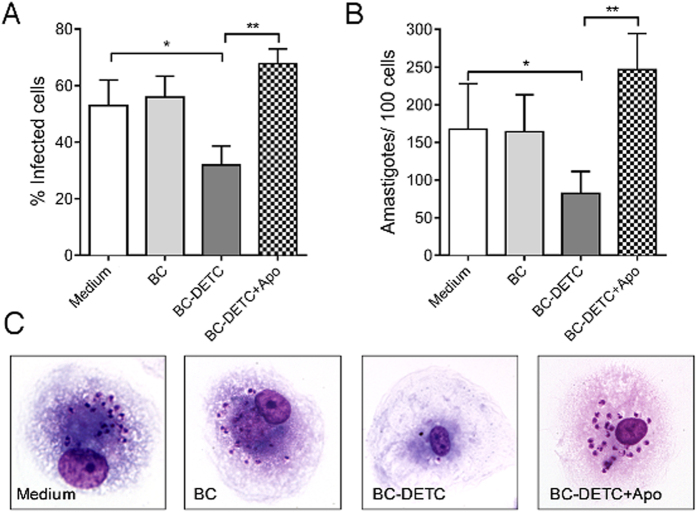
BC-DETC leishmanicidal effect on human macrophages infected with *L. braziliensis*. Macrophages were infected and were exposed to BC-DETC (IC_50_) (284.9 μg DETC/cm^2^) in the presence or absence of apocynin (APO) (20 μM). Cells were assessed for (**A**) the percentage of infected macrophages (**A** and **B**) for the number of amastigotes per 100 macrophages. (**C**) Representative photomicrographs of cultures shown in (**A** and **B**). Data are presented individually. *p < 0.05, **p < 0.01.

We extended our investigation to human macrophages infected with *L. braziliensis*. In these cells, the IC_50_ of BC-DETC was determined at 284.9 μg DETC/cm^2^. Upon exposure of human macrophages infected with *L. braziliensis* to BC-DETC (IC_50_), we observed a 50% reduction in the percentage of infected cells (Fig. 7A) and a significant reduction in the number of amastigotes (Fig. 7B), compared to unexposed control cultures. Presence of Apocynin, an anti-oxidant, reversed the killing effect, confirming that microbicidal action of BC-DETC on human infected macrophages is dependent on superoxide (Fig. 7A and B). Representative photographs confirmed the reduction in intracellular parasites in BC-DETC-exposed macrophages whereas control cultures performed with empty BC or with BC-DETC + Apocynin showed the opposite effect (Fig. 7C). Finally, the CC_50_ of BC-DETC was calculated at 2,312 μg/cm^2^. The selectivity index of BC-DETC was established at 8.11.

## Discussion

The main control strategy against leishmaniasis consists on treatment with leishmanicidal drugs that, despite their long use, present problems regarding cost, toxicity, side effects, and increasing number of resistance reports^21^. We previously demonstrated that intraperitoneal injection of DETC is a therapeutic alternative for CL chemotherapy^8^,^11^. Herein we extended on these findings employing a topical formulation consisting of BC membranes loaded with DETC. The use of a topical treatment poses advantages such as easy application, less side effects and lower toxicity due to lower dosage, therefore increasing adherence to treatment and probability of success.

BC is biocompatible and biodegradable^22^ and, as such, has been applied in different contexts^23^,^24^,^25^. SEM showed that DETC penetrated and dispersed onto BC surface, resulting in membranes with elevated flexibility and homogeneous distribution, corroborating results obtained in with ibuprophen^26^, diclofenac^27^ and caffeine^28^ -loaded BC. FTIR spectra showed absence of novel peaks during BC-DETC preparation. The DETC peaks at position 1673 and 1616 cm^−1^ were shifted to 1739 and 1630 cm^−1^, respectively, and these chemical interactions suggest the formation of a controlled release system in which the free molecules are rapidly released by diffusion through the membrane, while molecules that interact with the membranes are slowly released.

Exposure of murine macrophages to BC-DETC did not result in cytotoxicity, at the concentrations of 3.5 and 35 μg/cm^2^ corroborating earlier findings^29^,^30^,^31^ and supporting the biocompatibility of BC. Macrophages infected with *L. braziliensis* and exposed to BC-DETC displayed a significantly decreased infection rate, which was associated with down regulated SOD activity and increased superoxide levels. These results are in agreement with our earlier finding that SOD-1 activation contributes with *Leishmania* survival whereas SOD1 inhibition by DETC promotes parasite killing in a superoxide-dependent manner^8^,^11^. *L. braziliensis*-infected macrophages exposed to BC-DETC displayed a significant increase in cytokine (TNF, IL-6 and IL-10) and chemokine (MCP-1/CCL2) release. TNF activates macrophages to kill *Leishmania*^32^; it also induces CCL2^33^ and ROS release^34^,^35^. ROS but not nitric oxide (NO) has been associated with killing in *L. braziliensis*-infected human monocytes^36^. Herein, we did not detect NO upon exposure of infected macrophages to BC-DETC (data not shown). We suggest that, *in vitro*, DETC-mediated SOD1 inhibition increased superoxide levels, synergizing with an elevation in macrophage-activating cytokines that collectively contribute to *L. braziliensis* elimination.

We then evaluated the therapeutic potential of BC-DETC *in vivo*. Topical application of BC-DETC *L. braziliensisi* lesions significantly decreased ear thickness and parasite load at the infection site but not within draining lymph nodes. Treatment with 17-AAG, a HSP90-specific inhibitor, also did not decrease parasite load in distal sites despite having a clear leishmanicidal effect at the lesion site^37^. In experimental *L. braziliensis* infection, parasites persist within draining lymph nodes, possibly resulting from the presence of regulatory T cells (Tregs) that secrete IL-10 and counteract effector T cells^38^. In *L. major* infection, Tregs (CD25 + Foxp3+ T cells) regulate the *Leishmania*-specific effector response. A dynamic equilibrium between Tregs and effector cells is established maintaining long-term persistence of low numbers of parasites in the skin [(rev. in ref. 39]. This outcome is advantageous for the parasite since the host is capable of transmitting *Leishmania* to sand flies^40^, but is also induces long lived immunity against re-infection, an advantage to the host. Indeed, complete elimination of the parasites reduces resistance to infection^41^,^42^. We speculate that the inability of BC-DETC to reduce parasite numbers in draining lymph nodes associated with the presence of Tregs during *L. braziliensis* infection^38^,^43^ could have a similar impact. Importantly topical BC-DETC performed as well as intraperitoneal injection of DETC^11^, with respect to decreasing lesion size and parasite load at the infection site. Similar results were obtained with Dissulfiram, a carbamate derivative similar to DETC, and *L. major*-infected mice^44^ and Dissulfiram in combination with kanamycin and amoxicillin showed an additive effect against *L. major* promastigotes^45^. These findings reinforce the potential of a DETC-based topical formulation for CL treatment.

Topical application of BC-DETC significantly decreased in IFN-γ and TNF production by dLN cells, compared to controls, in accordance with the milder inflammatory reaction observed *in situ*. We suggest that, *in vivo*, DECT-mediated SOD1 inhibition increased superoxide levels, leading to parasite killing and thus diminishing the inflammatory stimulus (reflected in lower IFN-γ and TNF levels). Although IFN-γ and TNF are key molecules for macrophage activation and leishmania killing, excessive inflammation is related to tissue damage and the development of more severe clinical manifestations of CL^20^,^46^,^47^. In accordance, Dissulfiram suppressed TNF, NO and PGE_2_ release after LPS injection^48^ and it also significantly inhibited the release of LPS-induced metalloproteinases and TNF via an increase in superoxide release^49^.

Given that treatment options for CL are currently limited and that the number of refractory cases has increased; topical application of BC-DETC can be envisaged as part of a combination treatment. Advantages of a combination treatment include increased efficacy, less drug resistance, lower drug dosage and a general decrease in side effects^50^. Combination therapy can also hinder the appearance of monotherapy-resistant parasites^51^ and, as such, it is being pursued in recent studies using Tamoxifen as an alternative to treat CL^52^,^53^. Herein, topical BC-DETC was as effective as intraperitioneal Sb^v^ when we evaluated lesion development and parasite replication at the infection site.

Lastly, exposure of *L. braziliensis*-infected cells to BC-DETC (IC_50_) decreased the parasite load in a significant manner. Apocynin reverted this effect confirming the superoxide-dependent parasite killing in human cells exposed to BC-DETC. To date, clinical studies employing topical formulations have demonstrated efficacy in CL caused by *L. major*^54^,^55^ and *L. panamensis*^56^,^57^ but similar trials have not performed in areas of CL caused by *L. braziliensis*. Given the current limitations regarding leishmaniasis chemotherapy, BC-DETC topical bio-curative^58^, described in the present work, is an effective and translatable addition to the existing chemotherapies currently available for CL.

## Methods

### Ethics statement

Female BALB/c mice (6–8 weeks of age) obtained from the animal facility at Instituto Gonçalo Moniz, FIOCRUZ were maintained under pathogen-free conditions. All procedures were done following the local Ethics Committee on Animal Care and Utilization recommendations (CEUA IGM-FIOCRUZ-L-001/12). All experimental protocols were approved by CEUA IGM-FIOCRUZ. Peripheral Blood was obtained from healthy individuals (*n* = 6) recruited in the city of Salvador (Bahia state, Brazil). This research was conducted with the approval of the ethical committee of Instituto Gonçalo Moniz (IGM), Fundação Oswaldo Cruz (FIOCRUZ) (Salvador, Bahia, Brazil; CEP 177/2008) and Comissão Nacional de Ética em Pesquisa (Brazilian National Ethics Committee, Brazil). All methods were performed in accordance with the guidelines and regulations determined by CEP. Written informed consent was obtained from each participant. No minors participated in the study.

### Preparation of Bacterial Cellulose membranes containing DETC (BC-DETC)

Bacterial Cellulose (BC) membranes were produced as described^17^: wet bacterial cellulose membranes were obtained from cultivation of *Gluconacetobacter hansenii* (strain ATCC 23769). Cultures were incubated for 96 h at 28 °C in tray- containing medium (glucose 50 gL^−1^, yeast extracts 4 gL^−1^, anhydrous disodium phosphate 2 gL^−1^, heptahydrated magnesium, sulphate 0.8 gL^−1^ and ethanol 20 gL^−1^). After three days of incubation never-dried hydrated BC membranes were obtained. BC membranes were washed in 1% NaOH at 70 °C to remove bacteria and rinsed several times in water, until a neutral pH was reached. Membranes were weighed and water mass (~50%) was removed by pressure. Purified BC membranes (25 cm^2^ disks) were used for DETC incorporation. DETC (D3506, Sigma) solutions (87.5 ug/mL, 875 μg/mL and 8,759 μg/mL) were prepared and 1 mL of each DETC solution was applied to BC membranes. Samples were incubated for 2 h. BC-DETC membranes were dried at 37 °C in a ventilated oven for 24 hours. Before use, BC-DETC membranes (loaded with the equivalent of 3.5, 35 and 350 μg DETC/cm^2^) were cut in disks (4 mm diameter) and sterilized before use by ultraviolet radiation for 15 minutes.

### Physical characterization of BC-DETC membranes

Scanning electron microscopic (SEM) images were obtained in a field emission scanning electron microscope (FESEM, JEOL JSM-7500F) after covering samples with a thin carbon layer.

### Viability of murine macrophages exposed to BC-DETC

Bone-marrow derived macrophages (BMDM) were seeded at density of 3 × 10^5^ cells per well in 24-well tissue plates. BC-DETC membranes 4 mm disks (loaded with 3.5, 35 and 350 μg DETC/cm^2^) were placed within cell culture wells. Control cultures were incubated in medium alone or with empty BC (0 μg DETC/cm^2^). Cells were incubated at 37 °C, 5% CO_2_ for 48 h. Plates were then centrifuged for supernatant removal. The total number of viable cells was estimated by Trypan blue exclusion considering 200 cells per well, in at least 5 random fields observed by optical microscopy.

### Parasite culture

*L. braziliensis* promastigotes (strain MHOM/BR/01/BA788)^18^ were grown in Schneider’s medium (Sigma) supplemented with 100 U/ml penicillin, 100 mg/ml streptomycin, and 10% heat-inactivated FBS (all from Invitrogen) at 26 °C. Stationary-phase promastigotes were used in all experiments.

### Macrophage infection with *L. braziliensis* and exposure to BC-DETC

BMDM were obtained as described above. Cells were resuspended in DMEM medium supplemented with 100 U/ml penicillin, 100 mg/ml streptomycin, and 10% heat-inactivated Fetal Bovine Serum (all from Invitrogen) and seeded at a density of 3 × 10^5^ cells per well in 24-well culture plates. Monolayers received 3 × 10^6^ cellsl *L. braziliensis* promastigotes and were incubated at 37 °C in supplemented DMEM medium for 24 h. Infected macrophages were then washed to remove non-internalized parasites. BC-DETC membranes (loaded with 3.5 and 35 μg DETC/cm^2^) were placed within culture wells and plates were incubated at 37 °C/5% CO_2_. Control cultures were incubated in medium alone or with empty BC (0 μg DETC/cm^2^). After 48 hours, cells were extensively washed, fixed and stained with hematoxylin and eosin (H&E). The number of infected cells and of intracellular amastigotes were counted by optical microscopy in 200 macrophages. Cultures were performed in quintuplicate. Alternatively, infected macrophage were exposed to BC or BC-DETC as described, monolayers were extensively washed and the medium was replaced by 0.5 ml of supplemented Schneider medium. Cells were cultured for seven additional days at 26 °C, when number of viable promastigotes was determined using hemocytometer.

### Quantification of superoxide SOD activity and cytokines in culture supernatants

BMDM were seeded at a density of 1 × 10^6^ cells per well in 24-well culture plates and cells were infected with *L. braziliensis* as described above. To determine intracellular SOD1 activity, infected macrophages were exposed to BC-DETC membranes (3.5 and 35 μg DETC/cm^2^) or to empty BC (0 μg DETC/cm^2^), as described. Forty eight hours later, cells were homogenized in cold 20 mM HEPES lysis buffer (pH 7.2) containing 1 mM EGTA, 210 mM mannitol, and 70 mM sucrose. Preparations were centrifuged at 1500 × g for 5 min at 4 °C and cytoplasmic SOD1 levels were measured using Superoxide Dismutase Assay Kit II (Calbiochem), according to manufacturer’s instructions. To determine superoxide production, BC-DETC or empty BC were placed within wells containing infected cells for 48 h in presence of 0.5 mM hydroxylamine hydrochloride (Acros Organics). Superoxide was quantified in culture supernatants using Griess reagent^8^. Cytokine levels were determined in culture supernatants using the mouse inflammatory Cytometric Bead Array (BD Biosciences), following manufacturer’s instructions. Data were acquired using a FACSort flow cytometer (Becton Dickinson) and analyzed using FlowJo Software version 7.6.4.

### *L. braziliensis* intradermal infection and therapeutic scheme

BALB/c mice were inoculated intradermally with *L. braziliensis* promastigotes (10^5^ parasites in 10 μl of saline) using a 27.5-gauge needle^18^. Ear thickness (as a surrogate for lesion development) was recorded weekly using a digital caliper (Thomas Scientific). Three weeks after parasite inoculation mice were randomly assigned into two groups (mean ear thickness at onset of treatment 0,33 mm ± 0,01): one group was topically treated with BC-DETC disks (loaded with 35 and 350 μg DETC/cm^2^) and disks were placed onto developed CL lesions. The control group was treated with empty BC (0 μg DETC/cm^2^). BC-DETC and empty BC were covered with Tegaderm Film (1624 W 3 M Health Care) and membranes were replaced three times a week, for five consecutive weeks. Ear thickness (as a surrogate for lesion development) continued to be recorded weekly. Six weeks after infection, parasite load was determined using by limiting-dilution analysis, as described previously^59^. In combination experiments, mice were infected and monitored as described. Infected mice were randomly assigned into four groups (mean ear thickness at onset of treatment 0,32 mm ± 0,01). Mice were treated with Sb^v^ (Glucantime^®^, 100 mg/kg/day, intraperitoneal, five days a week, for five weeks) alone, Sb^v^ (i.p.) plus topical BC-DETC, topical BC-DETC or topical empty BC.

### Cytokine quantification

To evaluate the cellular immune response in mice infected with BC-DETC-treated animals, mice were euthanized at different time points and retromaxillar draining lymph nodes (LNs) were homogenized in supplemented DMEM medium. Cells (10^6^/ml) were stimulated in presence of *L. braziliensis* promastigotes (5 parasites: 1 cell) for 24 or 48 hours. Control cultures were left unstimulated. Cytokine levels in culture supernatants were determined by ELISA (eBioscience), according tomanufacturer’s instructions.

### Human macrophage infection with *L. braziliensis* and exposure to BC-DETC

Human monocytes were isolated from peripheral blood of six healthy donors through Ficoll gradient centrifugation and plastic adherence. Human macrophages were cultivated in supplemented RPMI medium. After differentiation for seven days, cells were re-suspended in supplemented RPMI and seeded at a density of 3 × 10^5^ cells per well in 24-well culture plates. Monolayers were infected with *L. braziliensis* (10 parasites:1 cell) for 24 hours. Infected macrophages were washed to remove non-internalized parasites and were treated with BC-DETC; control cultures were incubated in medium alone or with empty BC (0 μg DETC/cm^2^). Cultures were also performed in the presence of BC-DETC and Apocynin, an anti-oxidant (20 μM) (Sigma). After 48 hours, cells were extensively washed, fixed and stained with hematoxylin and eosin (H&E). The number of infected cells and of intracellular amastigotes were counted by optical microscopy in 200 macrophages.

To calculate the half-cytotoxic concentration (CC_50_), non-infected macrophages were exposed to BC-DETC at different concentrations (350, 700, 1400, 3500, 7000, 14000 and 21000 μg DETC/cm^2^). To calculate the half-maximal inhibitory concentration (IC_50_), infected macrophages were exposed to BC-DETC at different concentrations (35, 70, 350, 700 and 1400 μg DETC/cm^2^).

### Statistical analysis

Data are presented as the median ± interquartile range. To evaluate disease burden in mice, ear thickness of mice following challenge was recorded weekly for each individual mouse. The course of disease for experimental and control mice was plotted individually. Disease burden was calculated as the Area Under the Curve (AUC) obtained for each mouse. Differences among BC-DETC-treated versus control mice were tested by Kruskal-Wallis (non parametric) followed by Dunn’s multiple comparison test, for comparisons between three or more groups. Comparisons between two groups were performed by Mann-Whitney (non-parametric t-test). Analyses were conducted using Prism (GraphPad, V 5.0) and a p ≤ 0.05 was considered significant. An unbiased hierarchical cluster analysis using Ward’s method was performed to test whether a combination of cytokine levels could differentiate BC and BC-DETC-treated groups Median cytokine values (Log2) measured at different time points were calculated for each treatment scheme. Heat maps were built using JMP(V 10.0). The half maximal-inhibitory concentration (IC_50_) of BC-DETC on intracellular *L. braziliensis* amastigotes and the half-maximal cytotoxic concentration (CC_50_) of BC-DETC for human macrophages were determined from a sigmoidal regression of the concentration-responses curves, respectively, using Prism (GraphPad V. 6.0). The selectivity index of BC-DETC was calculated as the ratio between the CC_50_ for human macrophages and the IC_50_ for intracellular *L. braziliensis* amastigotes.

## Additional Information

**How to cite this article**: Celes, F. S. *et al*. DETC-based bacterial cellulose bio-curatives for topical treatment of cutaneous leishmaniasis. *Sci. Rep.* **6**, 38330; doi: 10.1038/srep38330 (2016).

**Publisher's note:** Springer Nature remains neutral with regard to jurisdictional claims in published maps and institutional affiliations.

## Supplementary Material

Supplementary Information
