# Supplementary material for: DETC-based bacterial cellulose bio-curatives for topical treatment of cutaneous leishmaniasis
Source: Sci Rep. 2016 Dec 6;6:38330. doi: 10.1038/srep38330 (PMC5138610; doi:10.1038/srep38330)
Supplement: Supplementary Information [file srep38330-s1.pdf]

DETC-based bacterial cellulose bio-curatives for topical treatment of cutaneous leishmaniasis

Fabiana S. Celes[a], Eliane Trovatti[b,c], Ricardo Khouri[a], Johan Van Weyenbergh[d], Sidney J. L. Ribeiro[b], Valeria M. Borges[a], Hernane S. Barud[b,c], Camila I. de Oliveira[a,e*]

Supplementary Material

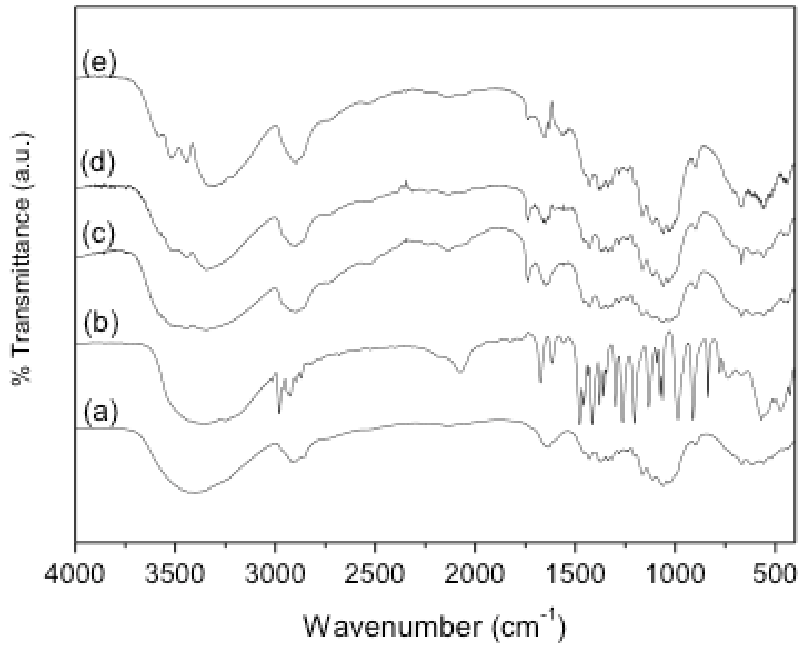

Supplemental Figure 1. Fourier transform infrared (FTIR) spectra of BC-DETC. *FTIR* spectra were obtained with a Perkin-Elmer spectrometer, model 2000, equipped with a single horizontal Golden Gate ATR cell. 64 scans were acquired in the 400-4000cm^{-1} range with a resolution of 4 cm^{-1}. Samples were milled and mixed with dried KBr in known proportions and pressed into pellets. Four samples of each membrane [control (empty), 3.5, 35 and 350 µg

DETC/cm^2] were analyzed. (a) BC, (b) DETC, (c-e) BC-DETC containing DETC at different concentrations.

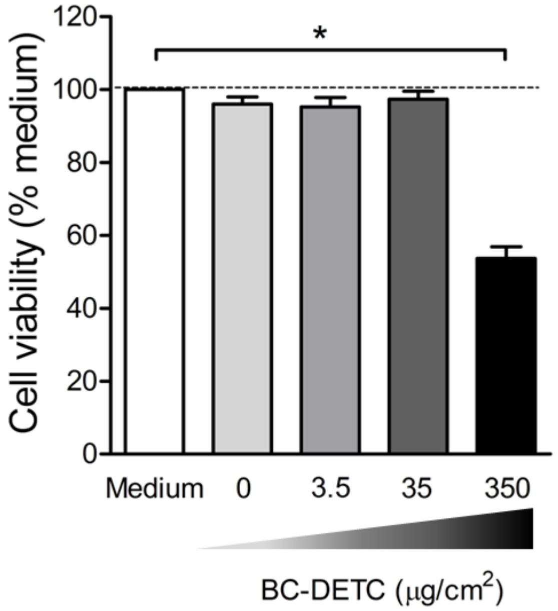

Supplemental Figure 2. Effect of bacterial cellulose membranes containing DETC (BC-DETC) on cell viability. Macrophages were exposed to empty BC or BC-DETC (3.5, 35 or 350 μg/cm^2). Cell viability was evaluated by Trypan blue exclusion assay after 48 hours treatment. Data are pooled from three independent experiments carried out in quadruplicates and are expressed in percent of untreated control (100%). *$p<0.05$

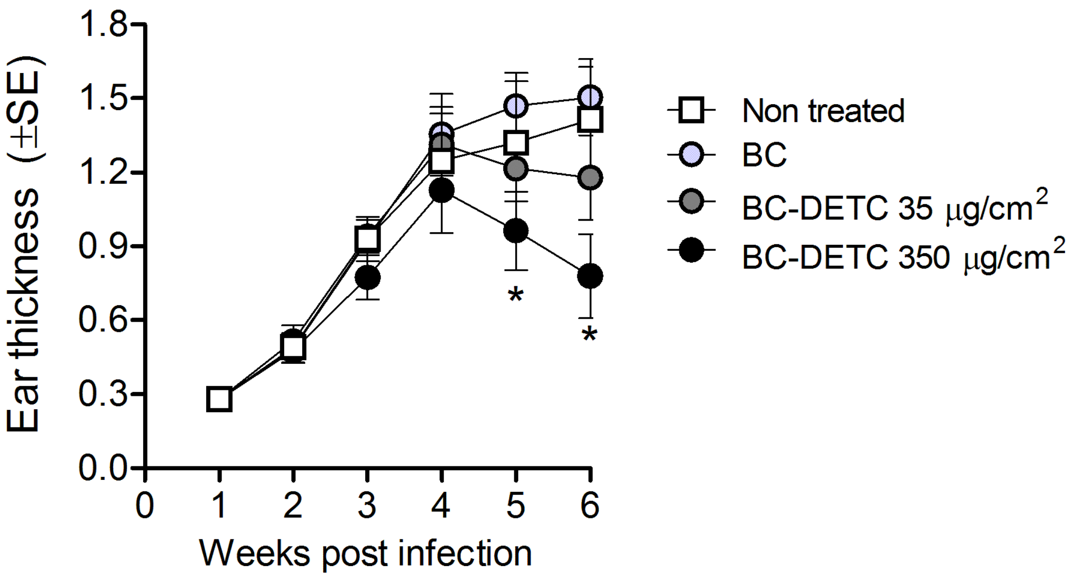

Supplemental Figure 3. CL development in mice infected with *L. braziliensis* and treated with BC-DETC. Mice were infected and three weeks later BC-DETC (at 35 and 350 ug/cm^2) was applied for three weeks (boxed area). Controls received empty BC. (A) Course of lesion development, measured weekly. Data are from one experiment, performed with four to six mice per group. *p <0.05.

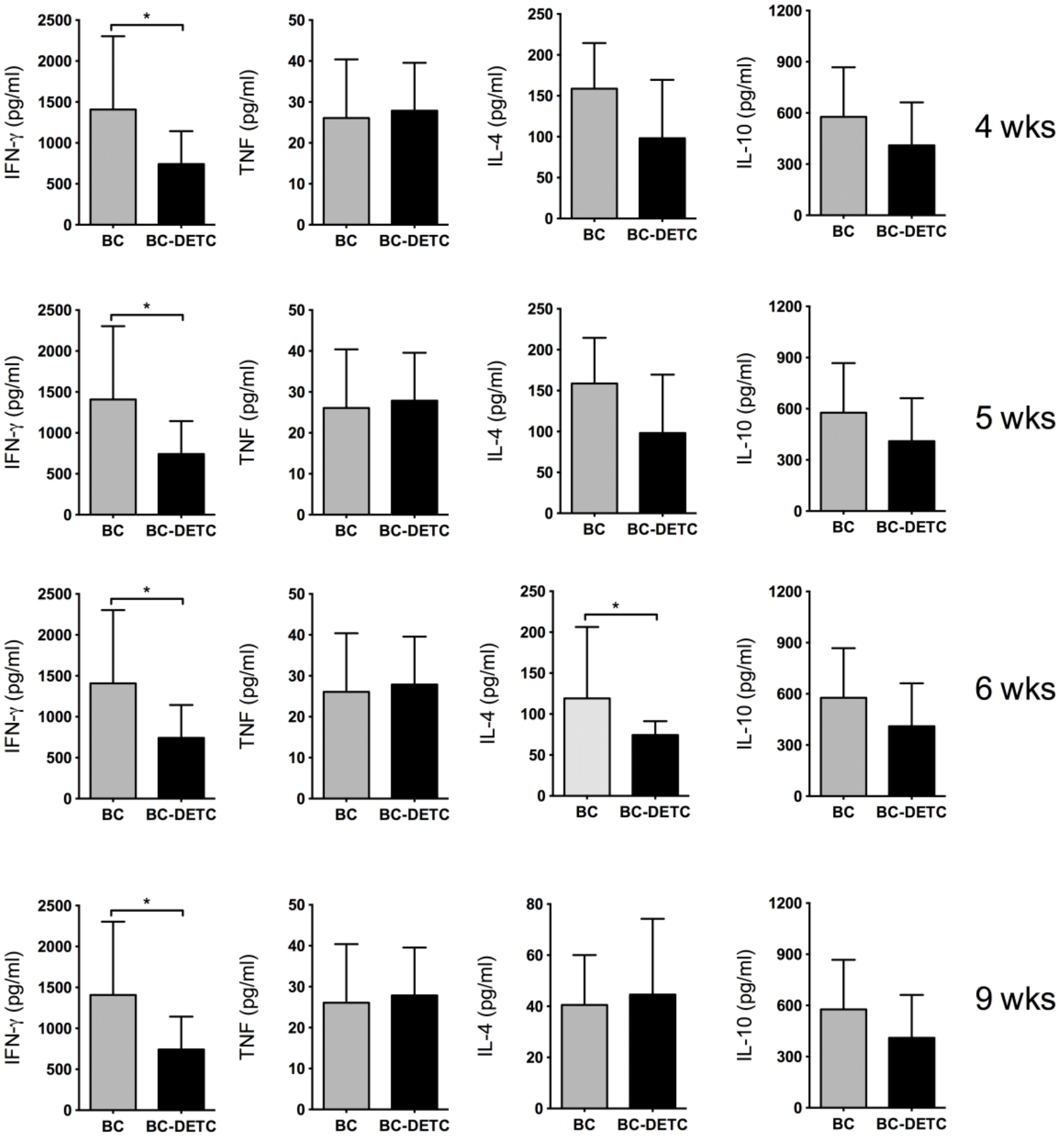

Supplemental Figure 4. Kinetics of cytokine production during treatment with BC-DETC. Mice were infected and three weeks later BC-DETC (at 350µg/cm^2) was applied for three weeks. Controls received empty BC. Draining lymph node cells were re-stimulated in vitro. Cytokines were quantified by ELISA at different time points. Data are from a representative experiment performed with six mice per group. *p <0.05.
